# Appalachia: Where Place Matters in Health

**Published:** 2006-09-15

**Authors:** Bruce Behringer, Gilbert H Friedell

**Affiliations:** Office of Rural and Community Health and Community Partnerships, Division of Health Sciences, East Tennessee State University; Markey Cancer Center, Lexington, Ky

## Health Disparities in Appalachia

Facts about health in the mountains of Appalachia have been slow to emerge. The formation of the Appalachian Regional Commission in the 1960s led to increased efforts to combat known precursors to poor health (e.g., low income, limited education, geographic isolation) (1). From New York's southern counties to the foothills of Mississippi, mountain counties were eligible to participate in various federal health programs because of their poor economic status. Critical private investments in health care occurred infrequently during the 1960s and still lag because of Appalachia's low population density and high percentage of residents without health insurance or with high-deductible plans.

Health care is largely organized, funded, and monitored through political channels. Public health programs, Medicaid funding, and vital statistics reports are organized by state. Health care service boundaries and health outcome patterns are not as clearly defined. Attempts to organize health status data across state lines within the formal boundaries of Appalachia proved to be a logistical and statistical nightmare (2). It was not until the National Center for Health Statistics (NCHS) produced national maps to display mortality rates that the truth about Appalachia's health status emerged (3). The maps proved statistically what residents knew intuitively: Appalachia, the place they called home, suffered disparately poor health compared with the rest of the nation.

The national computerization of health statistics and free Internet access to national and multistate databases have spurred additional exploration of health disparities in Appalachia. Recent studies have identified higher rates of cancer (4) and, in particular, cervical cancer (5); heart disease (6); and premature mortality (7) in the Appalachian regional population that spans state boundaries.

The articles in this issue of *Preventing Chronic Disease (PCD)* represent a new wave of studies that explore community-based explanations for Appalachian cancer issues by gathering and considering community perspectives on health and illness. The authors of these articles also share an implicit understanding of the relationship among people's health, their behavior, and their environment. This collection of research provides a view of some dilemmas faced by Appalachian health practitioners and advocates.

## Appalachian Dilemmas and Challenges

Why is addressing health improvement in Appalachia more difficult and different than it is with other populations and in other regions? The articles in this issue of *PCD* explain some of the dilemmas and challenges related to cancer prevention and treatment in this unique region.

### What people don't know about cancer

Several articles document that people lack facts about different types of cancer, are confused about differences among cancer screening procedures, and are not aware of publicly supported breast and cervical cancer screening programs. The data from the qualitative studies described in these articles provide depth and greater generalizability because they were collected in different communities and states. Focus group and survey participants reported that they gain most of their information about cancer from family, neighbors, and friends rather than from health professionals. Unfortunately, the information they receive often includes misperceptions of and dated knowledge about cancer treatments. The goal in Appalachia is to improve public cancer education while acknowledging and effectively using prevailing patterns of communication. The challenge is to tap local communication channels to disseminate accurate cancer information for communities while reinforcing that health professionals and health systems are important information sources.

### Tobacco as a leading risk factor for cancer and other chronic diseases

Wewers et al (8) document greater use of tobacco in Appalachian Ohio than in the rest of the United States, a finding that is unfortunately replicated across Appalachia. Community attitudes in the region are attributable in part to a deep-seated and historical economic dependence on tobacco growing and trading. The top five states in which tobacco represents more than 10% of total crops are located in the Appalachian region (9). Historically, families in the mountains remember tobacco as the "Christmas crop" because of the timing of payments received for their product from the tobacco auction. Local studies have found that even in more urban areas of Appalachia, 50% of primary care patients have some personal relationship with tobacco production, sales, or use (J Woodside, oral communication, June 2006). Cancer control strategies that address tobacco use in the region tread on difficult cultural and economic ground.

### The role of religion: fatalism or comforting factor? 

The importance of religion in Appalachian culture is well documented in these articles. Typically, authors have interpreted individuals' belief in "God's will" as evidence of a sense of fatalism toward health. However, an alternative interpretation is posed by this research. These studies find that Appalachians consider both their faith and the potential benefits of medical care when seeking solutions to health problems. Faith was not found to be a barrier to obtaining health care and is described as a comforting factor for people diagnosed with cancer. Behavioral theorists identify religion as an element of a person's "external locus of control" — an external circumstance that guides fate, luck, or behavior — in decision making about health (10). The authors of the studies in this issue of *PCD* point out that reliance on directions from health professionals is also present in Appalachia. To be effective in cancer control, health professionals must understand the balance of these influences and integrate this understanding in their goal to address cancer issues for individuals and the community.

### Low population density and service availability

Most of Appalachia is rural. Of the 13 states with counties located in the Appalachian region, 10 states have Appalachian counties with lower population density than their respective state averages (11). Appalachia is also characterized by many geographically isolated counties. Access to cancer care (12) is limited because of the region's history of a shortage of health care professionals and distance to referral centers from rural areas. However, small-town values of "pulling together" are exemplified in the cancer coalition article by Kluhsman et al (13) and described in the article by Coyne et al, which discusses sociocultural factors (14). The challenge in Appalachia is to build a set of cancer care services realistic for rural settings while ensuring access to highly specialized services at regional centers. Cancer control experts need to promote the value of cancer prevention, risk reduction, and screening services as important parts of cancer care that can be delivered by local providers in rural communities (15). Packaging these needed services may help rural residents see community cancer control as feasible and important, not as something available only through very expensive and distant cancer centers. Moreover, links between such centers and rural communities would clearly be mutually advantageous.

### Concerns about health and the environment

The influence of mountain culture on people's lives cannot be understated. Future qualitative studies will describe community members' concerns that traditional means of earning a living potentially have harmful effects on their lives. Of particular concern to rural communities are environmentally related causes of cancer. Concerns include toxic waste; unclean air; occupational exposures; and effluent from farms, mines, and factories that impact water quality. Environmental epidemiologists are constantly responding to community claims that cancer clusters have been identified. Appalachian residents are faced with an unenviable dilemma: they fear that environmental causes of cancer may be directly or indirectly related to the industries and jobs that allow them to remain in the mountains, which often prevents them from pursuing environmental action.

### Communication as the pivotal factor

Appalachians are characterized as proud, private, wanting to "take care of their own," and not accepting of charity. Our ongoing studies through the Rural Appalachian Cancer Demonstration Program, sponsored by the Centers for Disease Control and Prevention, have validated many of the points made in the articles in this issue. We have identified communication between patients and health professionals as instrumental in creating either trust or distrust between individuals and families and health care professionals and the health care system. Trust is the critical factor in individuals' acceptance of information and use of health care services, including screening and treatment for cancer. Personal trust is hard to gain but, once gained, hard to lose in Appalachia. Health professionals face the challenge of acknowledging these personal characteristics of Appalachians and using them to develop two-way communication about cancer. An additional challenge is to communicate public cancer messages outside of traditional health visits as well as find ways to effectively integrate messages about screening and prevention into traditionally busy practices and brief health care encounters.

## Conclusion

The mountains shape people's lives, both literally and figuratively. There is clearly a distinguishable Appalachian culture, and "place" is a prominent feature in that culture. Our cancer control studies have identified numerous cultural issues that influence cancer incidence, mortality, and cancer care in the region. Actions and beliefs in Appalachia are largely based upon discussion among community members about their experiences with disease and health care. Communication and use of care is influenced by skepticism, some distrust of health professionals, and fear of being taken advantage of by "the system." Residents report that poor communication between health professionals and patients further creates complications in health care delivery and represents a barrier to pursuing cancer screening, diagnosis, and treatment.

Those cultural issues undergird one final dilemma not addressed in the articles: the Appalachian regional population has lower income and poorer educational achievement and is older than the general U.S. population. These characteristics are generally seen as precursors to poorer health status. Yet location as a precursor of poor health has been reserved to states, generally southern, that frequently appear on "the worst" lists. Little attention has been paid to culturally defined geographic areas. Seven of eight Appalachians are white, and most nonwhite Appalachians live in southern Appalachian states. Comparisons between mortality rates among whites in Appalachia and whites in the United States as a whole had not previously been analyzed but became visually apparent on the NCHS maps. So, too, was the long-overdue comparison of Appalachia's black population mortality rates with national black mortality rates. Both sets of Appalachian mortality rates exceed national rates (7). The Appalachian disparities dilemma is that although poorer health outcomes in the mountains conform to popular regional beliefs, the disparities have not been recognized regionally or nationally.

Appalachians traditionally do not seek attention, and they try to manage their own problems. However, the geographic, health systems, and cultural issues that affect cancer in this region may be too large and complicated to address without significant external attention and assistance. The articles in this issue of *PCD* help shed light on, and give depth to, the dilemmas we face as public health practitioners in Appalachia.
